# Monitoring Temporal Trends in Internet Searches for “Ticks” across Europe by Google Trends: Tick–Human Interaction or General Interest?

**DOI:** 10.3390/insects13020176

**Published:** 2022-02-08

**Authors:** Per M. Jensen, Finn Danielsen, Sigurdur Skarphedinsson

**Affiliations:** 1 Department of Plants and Environmental Science, University of Copenhagen, Thorvaldsensvej 40, 1871 Frederiksberg, Denmark; 2 Nordic Foundation for Development and Ecology (NORDECO), Skindergade 23, 3rd Floor, 1159 Copenhagen, Denmark; fd@nordeco.dk; 3 Clinical Center for Emerging and Vector-borne Infections, Department of Infectious Diseases, Odense University Hospital, Winsloews vej 4, 5000 Odense, Denmark; s.skarphedinsson@rsyd.dk; 4 Research Unit of Infectious Diseases, Department of Clinical Research, Faculty of Health Science, University of Southern Denmark, Campusvej 55, 5230 Odense, Denmark

**Keywords:** *Ixodes ricinus*, Google Trends, tick–human contact, vector bone diseases

## Abstract

**Simple Summary:**

It is notoriously difficult to track the spread of vector-borne diseases, and to determine the underlying causes of change in disease occurrence. Importantly, public health representatives have limited access to information, which permit them to assess trends and changes in vector–human contact. Exposed individuals may, however, seek information on the Internet and thereby deliver input to a search record for the given vector. The variation in search-frequencies may reflect the variation in contact rates, but varying general interest could also influence the data. We here investigated records for search terms synonymous with “tick(s)”, and found that the records reflect the seasonal variation, which one would expect when these result from tick encounters. Albeit, variable use of search terminology suggest that these records should not be used to make comparisons between years.

**Abstract:**

Monitoring vector–human interaction is pivotal for assessing potential transmission rates of vector borne diseases and their associated public health impact. People often seek information following an insect bite in order to identify hematophagous arthropods, which in recent years often is done using Internet resources. Through this activity, a record of net searches is generated, which include information that reflect local human–arthropod interaction, e.g., for the common tick (*Ixodes ricinus*) in European countries. Such records could in principle provide low cost real-time monitoring data, if indeed Internet search activities adequately reflect tick–human interaction. We here explore Google Trends records for within-year and between-year trends, for four different Danish search terms for “tick(s)”. We further assess the relationship between monthly search-frequencies and local weather conditions (temperatures and precipitation from 2007 to 2016) in nine European countries. Our findings point to significant limitations in the records due to changes in search-term preferences over the given years. However, the seasonal dynamics are comparable among search-terms. Moreover, the seasonal pattern in search terms vary across Europe in tune with changes in temperature and precipitation. We conclude that, the within-year variation for given search-terms provide credible information, which systematically vary with local weather patterns. We are not convinced that these records merely reflect general interest. It will, however, require a more in-depth analysis by researchers that have specific insight into local language practices to fully assess the strength and weaknesses of this approach

## 1. Introduction

Changes in vector–host interactions can alter transmission patterns of pathogens, which negatively affect animal and human health [1]. The transmission varies with (i) human activity patterns; (ii) vector abundance; and (iii) the prevalence of infection in the vector population. Our understanding of the mechanisms, which regulate these three factors, rarely permit us to predict the overall effect of environmental change. Vector–host interaction is therefore typically assessed by surveillance/monitoring. Monitoring vector-human interaction is however associated with considerable challenges, as individuals often are unaware that they have been bitten by hematophagous arthropods [2]. Even when they are aware of this, they would be unlikely to report a frequently occurring “insignificant” event. The incidence or prevalence of infection/clinical illness is therefore often the only source of information, which reflect long-term changes in vector–human interaction [3]. These clinical records are unfortunately often of poor accuracy due to diagnosis-coding limitations and underreporting [4]. Continuously updated information on vector–human contact rates will provide a much-improved base for decision-making, which permits timely measures to reduce risk to human health from hematophagous arthropods.

The tick *Ixodes ricinus* is an important vector of infectious disease in humans in Europe. A large number of studies have been carried out in past decades, which allow an understanding of its biology, and the factors that govern its lifecycle and host-seeking activity. The species of most concern in Northern Europe is *Ixodes ricinus*. The list of relevant species also includes *Hyalomma marginatum*, *Dermacentor marginatus* and *Dermacentor reticulatus*, as well as *Rhipicephalus sanguineus* in Southern Europe. The European Centre for Disease Prevention and Control (ECDC) has compiled national records, which document their distribution [5]. More detailed maps have also been generated, which inform on the local distribution of ticks [6,7]. Albeit, few studies have assessed the temporal variation in tick bites, which reflect tick–human interaction, e.g., [8,9]. Long time-series are rare [10], as the cost of such monitoring activity is prohibitive. Citizen Science has increasingly been suggested as a solution to such monitoring challenges. It is argued that this type of monitoring have lower costs if/when the data are collected by unpaid volunteers, reported electronically, and compiled automatically [11,12]. A number of mobile phone-based tick monitoring solutions have been produced by companies and NGO’s e.g., [13,14]. These systems can deliver accurate data, when volunteers receive proper introduction to tick identification and a guide to a standardized report methodology [15,16]. The precision and spatial bias of the information will however depend on the size of the user-base and its distribution. Maintaining the base of informants such that the monitoring continues, may be a challenge. The viability of these schemes depends on the ongoing recruitment and active participation of people with a particular interest [17,18].

Records of web-search-term frequencies represents an alternative form of information, which may be classified as a “passive” citizen science scheme [19]. The strength of this approach lies in the sizable base of informants, which are recruited continuously. It does however have its own particular challenges [20], because the “informants” have limited insight into the subject, on which they are seeking information. The reservations that we may have in using Internet search records, due to undocumented behavioral patterns, are considerable (see Section 2.1). The potential benefits are however equally great, because this data source do not suffer the vulnerabilities of active citizen science schemes.

We here assess the credibility of Internet search records by investigating the temporal and geographic characteristics for Danish search terms synonymous with “tick(s)”, as recorded by Google Trends. We further assessed how search-frequencies varied with temperature and precipitation month-by-month across the European continent. Through these analyses, we take a first step in assessing whether Google Trends search-frequency data reflect tick–human interaction or simple general interest.

## 2. Materials and Methods

### 2.1. Regarding the Credibility of Search Term Records

A search for Google Trends records for the term “tick(s)” may, for a number of reasons, be inadequate. Internet searches may include flawed terminology, i.e., people search for information using a popular trivial name for ticks rather than the term “ticks”. Both trivial names and proper names may be used in singular and plural form, which may vary in usage between regions. Simple spelling mistakes would also affect the input, e.g., when individuals seek information on “tics” instead of “ticks”, and when people in their search exclude diacritics (e.g., ‘, ~, ^.) because it is faster to type. Temporal bias may also arise, because people are less likely to seek information on ticks late in the tick-season, if they had searched for information due to a tick bite earlier. Similarly, Internet searches performed due to later infections, could lead to a temporal misalignment.

We may in general terms consider that Internet searches reflect either (i) sporadic; (ii) general; (iii) proactive; or (iv) reactive interest. Sporadic interest is by definition sporadic and presumably linked to inspired moments or whims, which occur randomly. General interest should by definition be general and occur with fixed frequencies in every month of the year. Proactive interest is reflected in anticipatory action, which in this case permit individuals to identify ticks if they are encountered. Reactive interest follow encounters with ticks, which mean that either human or companion animals attracted a questing tick. Both events would provide relevant information on tick–human interaction, even though this is diluted with observations on animals. This dilution will be of little concern in northern Europe, but for more southern countries this dilution include additional tick species, such as *Rhipicephalus sanguineus* and *Dermacentor marginatus*, which have different host preferences and seasonal activity patterns. With this array of concerns, we suspect that Internet search records are unlikely to reflect tick–human interaction. Yet, studies have shown that the search frequencies provide reliable records on tick borne diseases [21,22] just as it has for infectious disease incidence in general [23,24]. Perhaps, search frequencies inform adequately on tick–human interaction as well.

Due to the given concerns, we chose to perform an initial examination, which first investigates the terminological (in)consistencies. Temporally diverging trends between tick-synonyms would reflect poorly on the credibility on all search terms. Second, we assessed the seasonal patterns in countries with different climates, because the varying weather patterns must impose differences in tick activity patterns. If all countries have similar patterns, then search frequencies reflect sporadic and/or general interest.

### 2.2. Google Trends Search Data Entry, Selection and Reports

When search terms are entered in Google Trends, the site–engine automatically display result for the last 12 months, day-by-day [25]. The search can then be modified to include particular contexts (ticks as search term, as animal, etc.), areas (nations, regions) and timespan (weeks, months and years). Further filtering can be chosen by limiting the search to specific categories or type of search (web, picture, shopping), and the outcome of the search is defined as much by these filters as the search-term itself. Comparisons between search terms are performed by adding another search-term, in which case the report will include information on both. All search-term reports are given as relative frequencies in integers with a maximum value of 100. This also applies when multiple search-terms are used, which reduce the data resolution when one search term has much higher frequency than the other(s). If a search term at one particular time-point has a single extreme frequency, all remaining frequencies are reduced to lower and more uniform frequencies. The temporal reporting base is adjusted automatically with the timespan, i.e., longer timespans generates monthly reports, while shorter timespans generate weekly reports, which combined with the automated frequency distribution makes it complicated to extract high data resolution reports over longer timespans. In the following, we retrieved data for single terms with a long time span (2007 to 2019), which yielded monthly reports.

### 2.3. The Influence of Search Terms in Records Pertaining to Denmark

The proper terms for ticks in the Danish language is “Flåter”, in singular: “Flåt”. It is however quite common that ticks are referred to as “Tæger”, in singular: “Tæge”, which we speculated would be used as frequently as “Flåter”. We suspected that the use of the proper term would be increasingly used as public-media have adopted the term “Flåter” in recent decades. We further suspected that the speed of change would differ between regions, due to differences in age structure in the human population and varying occupational patterns (a change in terms would be faster among young people who remain unaffected by work-related oral traditions). We collected the relative search frequencies for the four Danish search-terms and compared the variation in use in the period from January 2007 to December 2019. The search was conducted for the entire country and for the region of Southern Denmark. We expected that the long-term trend would be stable for the entire country, while the report would document a declining abundance of ticks in Southern Denmark, where an infectious disease recently led to a 50% reduction in the roe deer population [26]. This depletion of tick blood hosts was associated with a substantial local decline in Lyme Borreliosis cases, presumably because tick numbers declined.

### 2.4. Seasonal Characteristics and Their Association with Varying Weather Patterns across Europe

The relationship between weather and tick activity is quite complex and influenced by co-occurring changes in host abundance [27], which makes it difficult to predict details of the lifecycle: development, mortality, and host seeking activity, e.g., [28,29]. However, such insights are not required for assessing whether the frequencies vary systematically within the year. In fact, such analyses only inspect whether a given cohort of ticks become active and die out in accordance with ambient weather conditions [30]. For a given annual cohort, it can be expected that ticks become active in the spring when temperatures increase, which will appear earlier in Southern Europe than in the north [29]. It could also be proposed that the cohort will be spent quicker in warmer climates, and hence that the increase in search frequencies will come early and last for a shorter period in warm and dry regions. Last, we expect that tick population in continental climates would suspend activity during winter, while tick populations in the western part of Europe would be active throughout the year, such as permitted by varying degree of diapause [31].

We used Google Translate to identify a national term for “ticks” in Spanish (garrapatas), French (tiques), Norwegian (flått), Lithuanian (erkes, i.e., not erkės), Bulgarian (къpл**e**жи), Croatian (krpelji), Czech (klíšťata) but used the term “tick” in Irish, due to insufficient records for the Irish term: sceartáin. Search frequencies were extracted using the given term in the appropriate nation. For France, the search was limited to the region Pays de Loire to extract information from an area, which reflect the tick phenology in the Atlantic climate of Western France. A single search term will not inform on the total number of searches in each country, as there may be several synonyms. Foreign nationals and tourist will in all likelihood perform searches in their own language and the total number of searches can only be assessed by the inclusion of multiple search terms. The sum will however be biased because the number of tourist vary seasonally. They may also markedly inflate local population densities in areas, which typically have low population density. A single local language search term will not be vulnerable to such perturbations, but can only serve as an index.

We calculated simple statistics, which in simple terms characterized the within-year patterns. We calculated the skewness (a measure of symmetry relative to the normal distribution), kurtosis (whether the distribution is heavy or light tailed relative to the normal distribution), coefficient of variation (the amount of variation compared to the mean of the same data) for each year. We further identified the month of maximum search frequencies, the month of the steepest increase, and the month of the steepest decline. The given measures were calculated for each year, which then served as basis for calculating the mean skewness, etc. over the 10 y period. The monthly trends (increase and decrease) in search-frequencies was assessed by linear regression for the current and past two months (Excel slope functions with frequencies as *y*-axis date and month numbers fixed as 1–3). We then examined the relationship between monthly search frequencies and ambient weather conditions, with the expectation that temperatures and precipitation would capture the given variation [32]. For this purpose, corresponding monthly mean temperatures and precipitation for the period 1901 to 2016 was retrieved from the World Bank Group Climate Knowledge Portal [33]. The monthly relative frequencies were analyzed for the association between current mean monthly temperature (°C), current mean monthly precipitation (mm), previous month’s temperature, previous month’s precipitation and year, for logarithmic transformed frequencies. “Year” was entered as a categorical variable, which meant that the inter-annual changes, which might occur due to change in host abundance, changes in search terms, increase in public debate of tick-borne diseases, etc., were removed.

Descriptive statistics were extracted by mathematical functions in Excel. Statistical analyses were performed in SAS 9.4 (SAS Institute, Cary, NC, USA).

## 3. Results

### 3.1. The Influence of Search Terms

**Figure 1:**
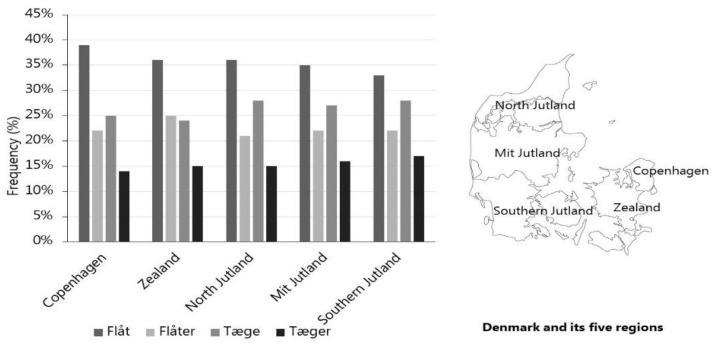
The reported relative search-frequencies for four different Danish tick terms: “flåter”, “flåt”, “tæger”, “tæge” in different regions of Denmark in the period 2007–2019.

**Figure 2:**
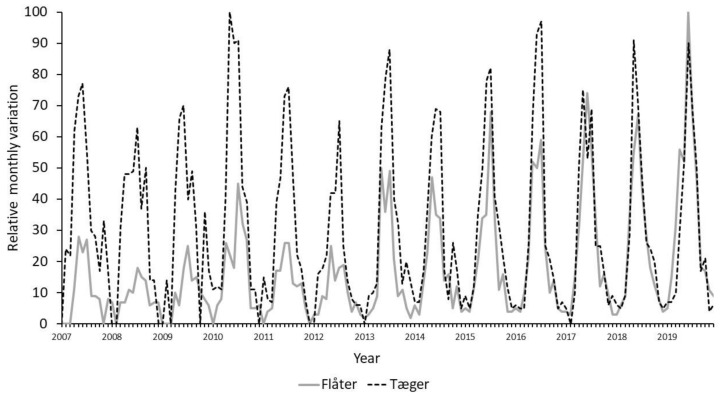
The monthly variation in two Danish tick search terms: “Flåter” and “Tæger” in the period of 2007 to 2019.

The four Danish terms for tick(s): flåt, flåter, tæge and tæger, were all commonly used in Denmark, but the search term “flåt” appears to be slightly more frequently used than other terms across all Danish regions (Figure 1). This pattern was, however, not stable in time as the term “flåter” gained in use over the years relative to the use of “tæger” (Figure 2).

Thus, the trends over the years were positive for the term “flåter”, while there was no particular trend for “tæger” (Table 1). The search term “flåt” also increased in frequency over the years in Southern Denmark. In contrast, the term “tæger” declined in frequency. The four terms were, however highly correlated, suggesting that they all exhibit similar seasonal variation. The correlations were stronger on the national scale than on the regional scale (Table 1).

### 3.2. Seasonal Characteristics

**Figure 3:**
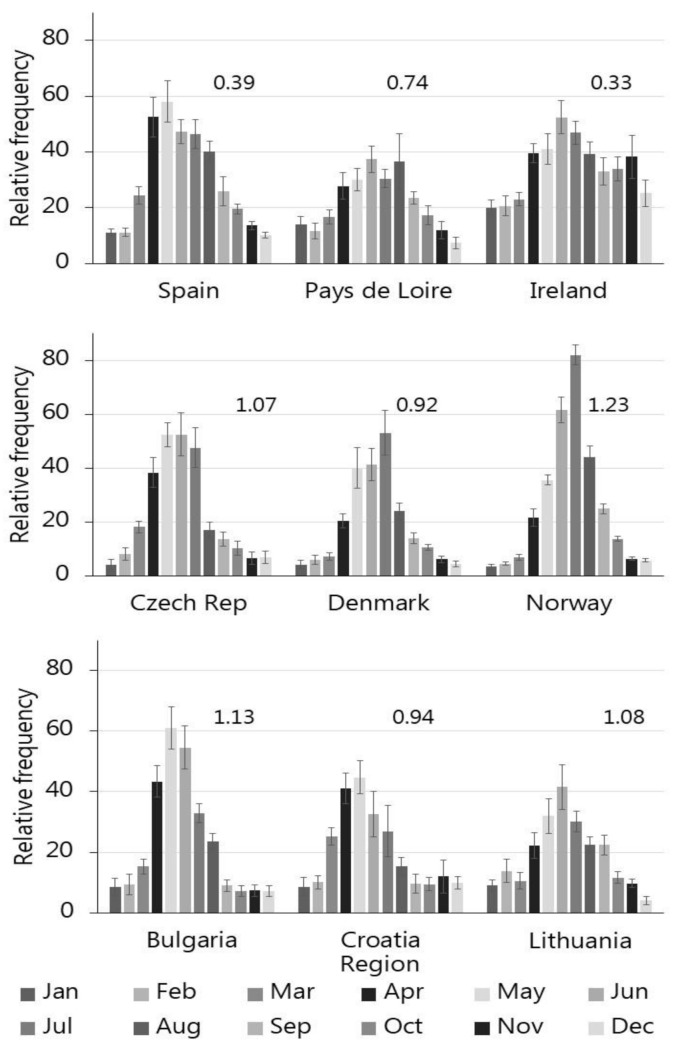
The relative monthly variation in tick search-terms in nine different countries/region in Europe in the period 2007 to 2019 (Mean ± SE).

Within-year search frequencies had a positive skew, i.e., there were a majority of months with lower frequencies and relatively fewer with high frequencies (Table 2, Figure 3). The lowest skewness were found in coastal regions (Spain, Pays de Loire and Ireland). Similar geographic patterns was noted for within-year kurtosis and coefficient of variation (Table 2), suggesting that these descriptive statistics changed systematically with the geo-graphical location (Figure 3).

The within-year mean maximum annual frequencies fell early in warm and continental climates (Table 2, Figure 3). In Croatia, Bulgaria and Spain maxima occurred in early to mid-May, while the maximum were in late May in Lithuania. In the cooler oceanic climate in Pay de Loire, Denmark and Norway, maxima fell in June, while in Ireland the maximum fell in July (Table 2). The maximum increase in search frequencies fell ½ to 1½ month earlier. All countries had the greatest decline in searches between early August and early September.

**Figure 4:**
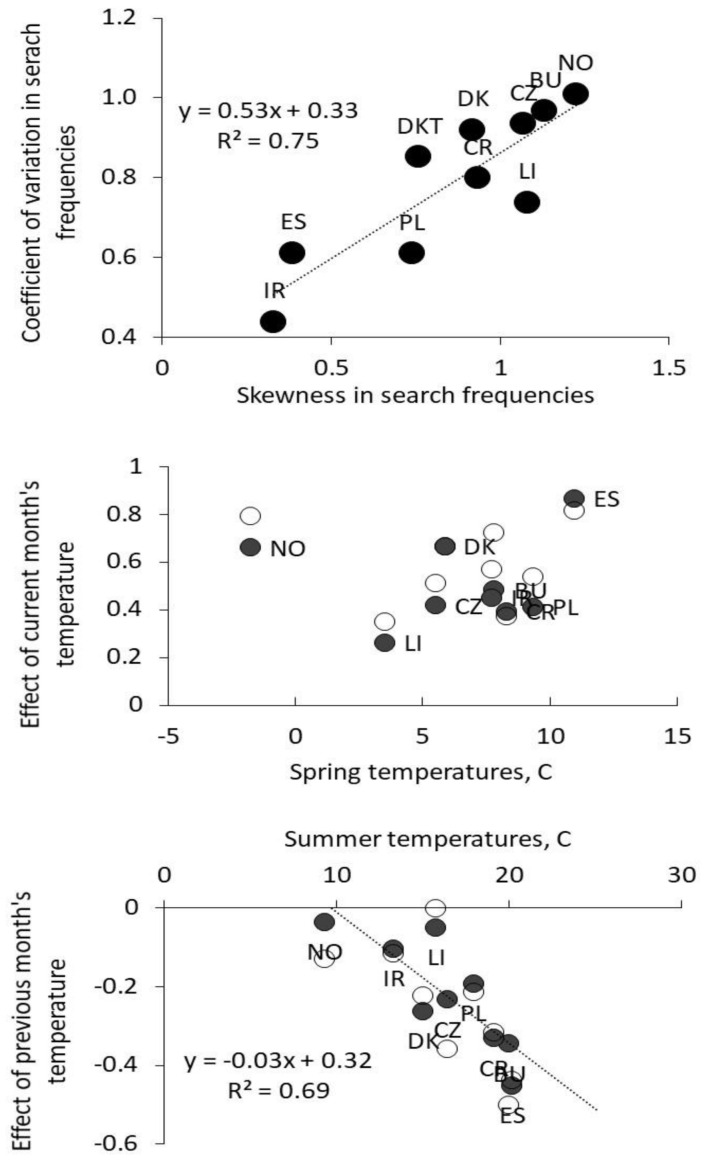
The relationship between skewness and coefficient of variation (**top**). Plot of estimates for the effect of temperatures in current month vs. mean temperature −0.5 sd (spring temperature: **middle**) and plot of estimates for the effect of temperatures in previous month vs. mean temperature + 1 sd (summer temperature: **below**).

The temperature of current month were positively correlated with search-frequencies for all regions. The impact of previous month’s temperature was strongly negative for regions with high temperatures (Spain, Bulgaria and Croatia), significantly negative in Pays de Loire, but had no effect in Ireland, Lithuania and Norway. The amount of precipitation only affected search frequencies in two countries: positively in Spain, while a negative impact was noted in Norway. There seems to be little relationship between the effect of current-month’s temperature and spring temperatures (mean monthly annual temperature − 0.5 sd), while the negative effect of previous month’s temperature is clearly associated with summer temperatures (mean monthly annual temperature + 1 sd; Figure 4).

## 4. Discussion

Our analysis clearly demonstrates that the Danish terms: “flåt, flåter, tæge and tæger” are used unevenly in Danish regions. The inter-annual trends appear to be highly sensitive to gradual changes in search term preferences, and the differences are of such scale that the term defines the trend (Figure 2). The development in the search-term “flåter” likely reflect the increasing adoption of this term, reflecting its increasing use in public media. We also note within-year differences between search-terms as “tæger” more often show two minor annual peaks in search-frequencies, while “flåter” typically has one. Possibly, this reflects that “flåter” mostly is used for nymphs, while “tæger” is used for all instars found on companion animals i.e., people get bitten by “flåter” and domestic animals by “tæger”. The descriptive statistics and analyses of the two set of records does however not support the notion, that these differences are relevant for assessing the seasonal patterns in tick search terms (Table 1, Table 2 and Table 3). We suspect that these comments are valid for all countries, but did not pursue this further, as such assessments requires a profound understanding of the national and local terminology and traditions. The linguistic challenges may however be of greater importance in countries that encompass multiple languages and a more diverse cultural base than Denmark, such as Austria, Switzerland and Belgium.

While the data appear to be reasonably robust across terms, we remain convinced that deviations from the true tick–human interaction arise when searches appear with a lag of days or weeks, or perhaps even months, when the interest arise from a suspicion of infection due to past tick bites [34]. There is no actual documentation of the bias that later occurring clinical illness induce, but it would be dismissive to ignore these and their impact on daily and weekly search-frequencies. Their impact on within-year patterns should however be relatively modest as few tick bites result in clinical infections [35], and because the lag mostly would be significantly shorter than a month (1–2 weeks, [34]).

For Denmark, the within-year patterns in search-frequencies continues the seasonal pattern of contacts to the Danish Pest Infestation Laboratory in the period 1948 to 2001 [36]. The within-year patterns in search-frequencies across Europe are in general terms comparable to this record as all records show a unimodal pattern, with peaks in search frequency between May and July according to the south-north gradient (Table 2, Figure 3). Moreover, the descriptive statistics informs on distinct differences between various parts of Europe such that the seasonal skewness, kurtosis and coefficient of variation differs systematically along the east–west gradient (Figure 3), which conform to the expected difference in within-year patterns across Europe [28,29].

The seasonal pattern in search frequencies are clearly associated with the regional temperature and precipitation. The relationship is not entirely systematic, as there seems to be no relationship between the size of the impact and the current temperatures (Figure 4). It could be suggested that the impact would be higher in cooler regions when ticks experience an increase in temperatures from below zero degrees. We may speculate that estimates for Southern European countries are biased due to the occurrence of other tick species, such as *Hyalomma marginatum*, *Dermacentor marginatus*, *Dermacentor reticulatus* and *Rhipicephalus sanguineus*. A varying degree of diapause may also intervene and cloud such straightforward response patterns [27,28,31]. The negative impact of past month temperature is however clearly connected to ambient summer temperatures (Figure 4).

**Figure 5:**
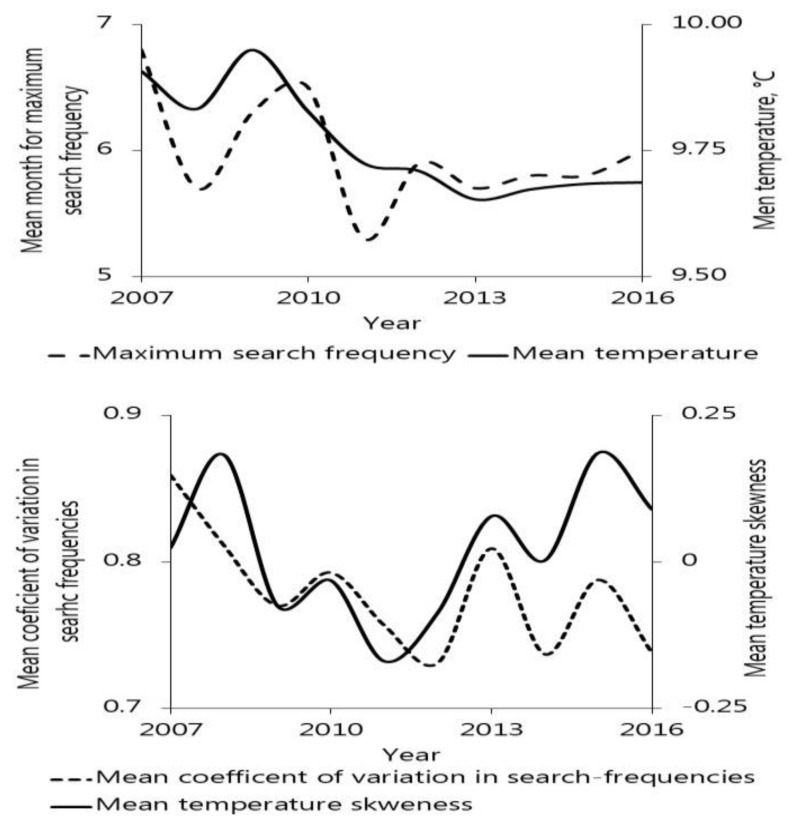
An illustration of how shifting within-years characteristics can be used to inform on the changes that occur over longer timespans. The inter-annual trends in mean month of maximum search activity vs. mean temperature (**top**) represents a conventional approach. The plot showing the coefficient of variation vs. temperature skewness for all nine regions in the period 2007 to 2016, includes an unconventional interpretation/representation of temperatures. Mean values are based on the nine countries.

With this, we conclude that search frequencies adheres closely to varying weather patterns such as we would expect when searches results from tick encounters. It is not possible to determine whether the searches were prompted by tick occurrences on humans or companion animals. Neither can we dismiss that some searches were proactive, prompted by media attention. We find it unlikely that sporadic or general interests would capture such relationships, and tentatively conclude that Google Trends data might be useful as proxy for tick–human interactions. In exploring this potential, emphasis should be placed on the systematic changes in within-year patterns, because they unlike the inter-annual variation in search frequencies, will be relatively unaffected by changes in terminology. Turning within-year characteristics into meaningful indices, which relate reliable inter-annual changes in environmental conditions may call for unconventional methods, e.g., by aligning the characterization of between-year temperatures and precipitation with the within-year assessment of search-frequencies (Figure 5). However, this should be a minor mathematical challenge for any agency that wishes to monitor tick–human encounters in real-time or do periodical reviews of tick-human interactions and the risk for tick-borne diseases.

This study is however only an initial appraisal. Further steps to validate Google Trends data should explore the impact of COVID-19 lockdown curfews on observed frequencies (preferably in a before–during–after design). The type of lockdown (with and without curfews), should yield significant effect. It would also be of interest to investigate whether seasonal characteristics deliver information on the local occurrence of tick-borne pathogens, since the search frequencies also could inform on the transmission of infectious disease between ticks and reservoir hosts.

## Figures and Tables

**Table 1:** Rank-correlation coefficients for four Danish tick terms and their trend over time (year) on Google Trends. The analyses include the years 2007 to 2019 (*n* = 156). *: *p* < 0.05, ***: *p* < 0.001.

Denmark Nationwide				
	Flåt	Flåter	Tæge	Tæger
Year	0.32 ***	0.30 ***	0.16	−0.08
Flåt		0.87 ***	0.88 ***	0.77 ***
Flåter			0.86 ***	0.77 ***
Tæge				0.81 ***
Southern Denmark				
Year	0.35 ***	0.32 ***	0.18 *	−0.19 *
Flåt		0.50 ***	0.63 ***	0.44 ***
Flåter			0.49 ***	0.36 ***
Tæge				0.57 ***

**Table 2:** Descriptive statistic for monthly tick-search-term frequencies in each of the 10 years (mean ± sd) covering the period 2007 to 2016 in nine European countries. The month of the largest increase and decrease in search frequencies was assessed by linear regression over the current and past two months and month where after the maximum and minimum slope was identified or each year.

Country/Region	Variable	Mean	SD		Mean	SD
Spain	Month of maximum	5.00	0.82	Skew	0.39	0.19
(14.0 ± 6.1 °C)	Month of largest increase	4.30	0.48	Kurtosis	−1.57	0.34
	Month of largest decrease	8.10	0.99	Coef. of variation	0.61	0.05
Pay de Loire	Month of maximum	6.60	1.51	Skew	0.74	0.76
France	Month of largest increase	6.10	1.73	Kurtosis	1.07	1.53
(12.2 ± 5.7 °C)	Month of largest decrease	9.30	1.49	Coef. of variation	0.61	0.24
Bulgaria	Month of maximum	5.40	0.70	Skew	1.13	0.67
(11.9 ± 8.1 °C)	Month of largest increase	4.80	0.63	Kurtosis	1.19	2.32
	Month of largest decrease	8.10	0.99	Coef. of variation	0.97	0.12
Croatia	Month of maximum	4.80	0.85	Skew	0.94	0.54
(11.9 ± 7.2 °C)	Month of largest increase	4.10	1.20	Kurtosis	0.56	1.30
	Month of largest decrease	7.40	0.84	Coef. of variation	0.80	0.09
Czech Rep	Month of maximum	5.50	0.85	Skew	1.07	0.34
(9.2 ± 7.3 °C)	Month of largest increase	4.70	0.67	Kurtosis	0.06	1.44
	Month of largest decrease	8.00	0.94	Coef. of variation	0.93	0.13
Denmark	Month of maximum	6.20	0.92	Skew	0.92	0.60
(8.9 ± 6.2 °C)	Month of largest increase	5.70	0.95	Kurtosis	0.41	1.44
(Flåter)	Month of largest decrease	8.60	0.70	Coef. of variation	0.92	0.17
Ireland	Month of maximum	7.20	2.25	Skew	0.33	0.74
(9.6 ± 3.7 °C)	Month of largest increase	5.60	2.07	Kurtosis	−0.39	0.55
	Month of largest decrease	9.10	0.99	Coef. of variation	0.44	0.09
Lithuania	Month of maximum	5.80	0.92	Skew	1.08	0.63
(7.6 ± 8.1 °C)	Month of largest increase	5.20	0.79	Kurtosis	1.73	2.31
	Month of largest decrease	8.60	1.43	Coef. of variation	0.74	0.10
Norway	Month of maximum	6.90	0.32	Skew	1.23	0.21
(1.9 ± 7.3 °C)	Month of largest increase	6.30	0.82	Kurtosis	0.65	0.87
	Month of largest decrease	9.10	0.32	Coef. of variation	1.01	0.10
Denmark	Month of maximum	6.40	0.84	Skew	0.76	0.37
(Tæger)	Month of largest increase	4.70	1.06	Kurtosis	−0.59	0.58
	Month of largest decrease	8.80	0.63	Coef. of variation	0.85	0.08

**Table 3:** Association between the frequency of online searches of tick in the national language and temperatures and precipitation in the current and previous month (2007–2016, *n* = 120).

Country/Region	Variable	Estimate	SE	Wald-Chisq	*p* > Chi	AIC
Spain	Current temperature	0.864	0.088	96.81	<0.0001	865
(14.0 ± 6.1 °C)	Current precipitation	0.015	0.007	4.85	0.027	
	Previous temperature	−0.452	0.063	52.22	<0.0001	
	Previous precipitation	0.014	0.007	3.86	0.049	
Pay de Loire	Current temperature	0.411	0.060	47.58	<0.0001	868
France	Current precipitation	0.004	0.006	0.37	0.54	
(12.2 ± 5.7 °C)	Previous temperature	−0.193	0.052	13.88	0.0002	
	Previous precipitation	0.011	0.006	2.75	0.097	
Bulgaria	Current temperature	0.482	0.055	77.83	<0.0001	910
(11.9 ± 8.1 °C)	Current precipitation	0.008	0.006	1.62	0.20	
	Previous temperature	−0.345	0.047	54.00	<0.0001	
	Previous precipitation	0.004	0.006	0.40	0.52	
Croatia	Current temperature	0.392	0.053	55.08	<0.0001	847
(11.9 ± 7.2 °C)	Current precipitation	0.000	0.004	0.00	0.99	
	Previous temperature	−0.332	0.051	42.69	<0.0001	
	Previous precipitation	−0.001	0.004	0.04	0.83	
Czech Rep	Current temperature	0.417	0.055	58.32	<0.0001	873
(9.2 ± 7.3 °C)	Current precipitation	0.010	0.007	2.18	0.13	
	Previous temperature	−0.233	0.046	25.55	<0.0001	
	Previous precipitation	−0.009	0.007	2.06	0.15	
Denmark	Current temperature	0.663	0.076	76.70	<0.0001	779
(8.9 ± 6.2 °C)	Current precipitation	−0.002	0.006	0.09	0.75	
(Flåter)	Previous temperature	−0.263	0.062	18.22	<0.0001	
	Previous precipitation	−0.007	0.006	1.31	0.25	
Ireland	Current temperature	0.449	0.092	23.69	<0.0001	943
(9.6 ± 3.7 °C)	Current precipitation	0.000	0.004	0.00	0.99	
	Previous temperature	−0.104	0.082	1.63	0.20	
	Previous precipitation	−0.001	0.004	0.12	0.72	
Lithuania	Current temperature	0.260	0.040	42.10	<0.0001	836
(7.6 ± 8.1 °C)	Current precipitation	−0.005	0.007	0.46	0.49	
	Previous temperature	−0.051	0.039	1.65	0.19	
	Previous precipitation	−0.007	0.007	0.94	0.33	
Norway	Current temperature	0.659	0.075	77.04	<0.0001	816
(1.9 ± 7.3 °C)	Current precipitation	−0.027	0.007	15.06	0.0001	
	Previous temperature	−0.037	0.052	0.51	0.47	
	Previous precipitation	−0.016	0.008	4.38	0.036	
Denmark	Current temperature	0.617	0.072	73.81	<0.0001	922
(Tæger)	Current precipitation	0.001	0.006	0.02	0.86	
	Previous temperature	−0.260	0.061	18.33	<0.0001	
	Previous precipitation	−0.007	0.006	1.36	0.24	

## Data Availability

The data is available from Google Trends and the World Bank Group.
